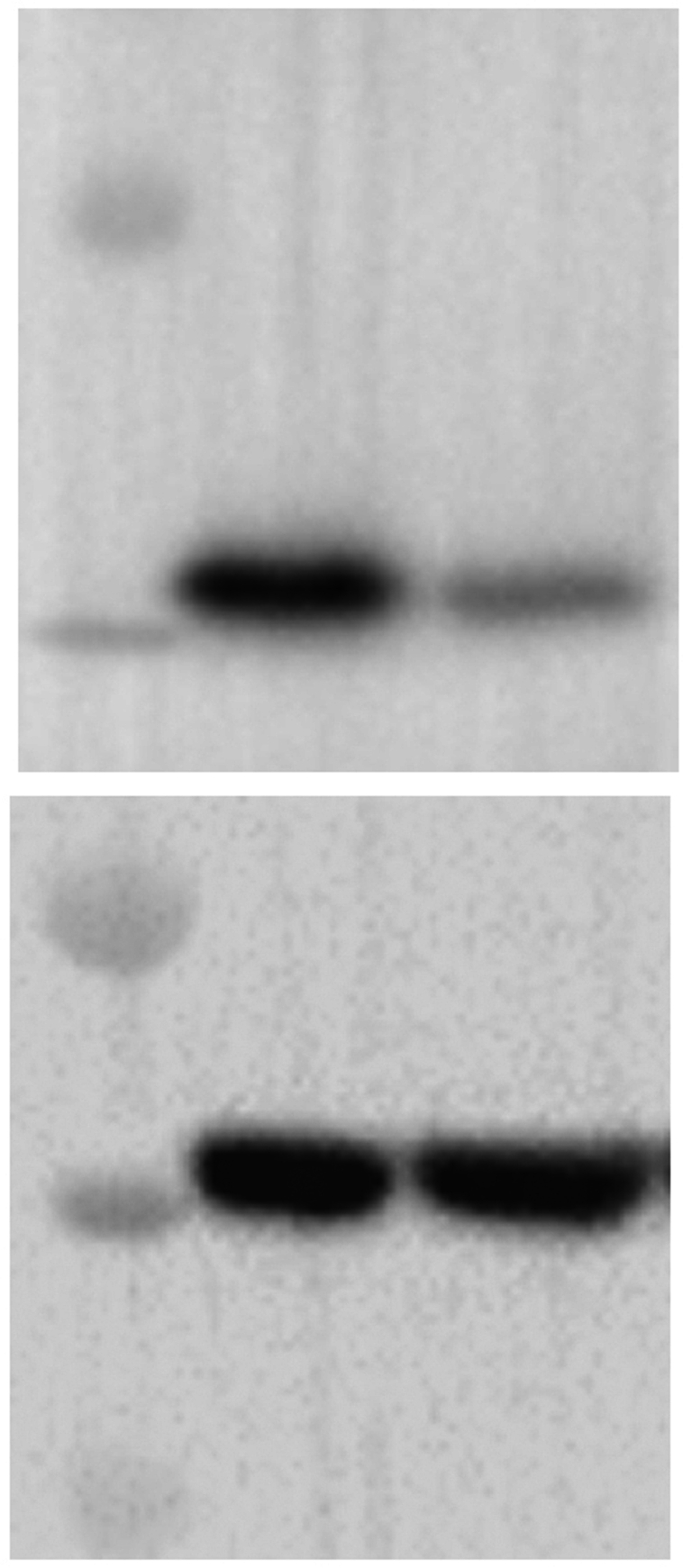# Quantitative proteomics assay reveals G protein-coupled receptor kinase 4-induced HepG2 cell growth inhibition

**DOI:** 10.1016/j.heliyon.2024.e29514

**Published:** 2024-04-12

**Authors:** Yunxiu Luo, Jing Yang, Yan Wang

**Affiliations:** a Hainan Cancer Hospital, Affiliated Cancer Hospital of Hainan Medical University, Department of Radiotherapy Oncology, Haikou, 570311, China; b Hainan Clinical Research Center for Hepatopathy and Liver Critical Illness, Haikou, 570311, China; c Guilin Medical University, Center for Science Research, Guilin, 541004, China; d Central South University, The Second Xiangya Hospital, Department of Surgery, Changsha, 410011, China

**Keywords:** G protein-coupled receptor kinase 4, Hepatocellular carcinoma, HepG2 cells, PPAR signaling pathway, Quantitative proteomics

## Abstract

**Background and aim:**

To investigate the biological effects and putative biological mechanism of G protein-coupled receptor kinase 4 (GRK4) on HepG2 cells.

**Materials and methods:**

Cell proliferation, cycle, and apoptosis were evaluated by Cell Counting Kit-8 and flow cytometry (FCM) in HepG2 cells infected with either the GRK4-overexpressing lentivirus vector (OE) or the negative control lentivirus vector (NC). The protein profiles and differentially expressed proteins (DEPs) of the OE and NC cells were analyzed and compared using the quantitative proteomics technique, and their function, expression, and probable mechanism were investigated using bioinformatic assays and parallel reaction monitoring (PRM).

**Results:**

HepG2 cells that received the OE grew more slowly than those that received the NC. FCM revealed that, when compared to the NC cells, the OE cells had undergone S-phase cycle arrest, and neither the OE nor NC cells underwent apoptosis. Among the 7006 proteins that were identified by quantitative proteomics, 403 DEPs were examined based on the filtering parameters, with the expressions of 135 being downregulated and 268 being upregulated. In addition to being involved in the peroxisome proliferator-activated receptor (PPAR) signaling pathway, the DEPs were implicated in the biological processes of cell proliferation, cycle, and metabolism. PRM verified the expressions of DEPs that were connected to the PPAR pathway.

**Conclusions:**

This study shows that GRK4 prevents HepG2 cells from proliferating and causes cell cycle arrest in the S-phase, while the PPAR pathway is involved in the regulation of HepG2 cells via GRK4.

## 1 Introduction

Hepatocellular carcinoma (HCC) is one of the primary causes of the increasing cancer burden, with 905,677 new cases and 830,180 HCC deaths reported globally in 2020 [1]. Innovative comprehensive therapies have contributed to an improved outcome, with the number of fatalities in China expected to be around 412,216 in 2022 [2]. The majority of deaths were caused by a failure to diagnose at an early asymptomatic stage, which is partly due to the scarcity of accurate predictors. Symptomatic cases may be detected early through thorough preclinical screening, whereas asymptomatic cases necessitate the discovery of novel biomarkers [3]. Reliable, effective biomarkers can improve therapy assessment and prognosis prediction. The present emphasis of the HCC research is a desperate need for new biomarkers.

G protein-coupled receptor kinases (GRKs) phosphorylate G protein-coupled receptors (GPCRs) that are triggered by extracellular ligand signals, and GRKs promote homologous desensitization of GPCRs by recruiting β-arrestin [4,5]. GRKs are serine/threonine-directed protein kinases with seven members from three families. GRK1 and GRK7 are both expressed in the retina and govern photoreceptor function in cones and rod cells via phosphorylating rhodopsin; hence, they are classified as both a visual and a rhodopsin family. The remaining five isoforms are members of a non-visual family. The β-adrenergic receptor family includes GRK2 and GRK3, which are extensively expressed in mammalian tissues and are controlled by the β-adrenergic receptor. The GRK4 family includes GRK4, GRK5, and GRK6. GRK5 and GRK6 have a wide distribution and varied functions. However, GRK4 expression is limited to specific types of tissues, such as the testis, particularly the fetal testis; brain; kidney; myometrium; and others [[4], [5], [6]]. The structure of GRK4, on the other hand, is quite similar to that of its family member GRK6, yet its function is entirely distinct. Most studies have focused on GRK4's role in hypertension management, with few demonstrating GRK4's role in neuron function transmission via γ-aminobutyric acid desensitization [7] and the maintenance of endocrine function via luteinizing hormone/follicle-stimulating hormone [8]. GRK4 is also involved in the regulation of the M2 muscarinic receptor and the endogenous calcium-sensitive receptor [5,[9], [10], [11]]. Importantly, several papers have reported the role of GRK4 in the pathogenesis of breast cancer [[12], [13], [14]]. We recently discovered that GRK4 was demonstrated to be associated with the prognosis of HCC patients [15], and patients with a high expression of GRK4 in their HCC tissues fared better in terms of survival than those who did not [15]. These findings indicated that GRK4 may play a negative role in the development of HCC, although the exact process was unknown. The current work investigates the potential molecular processes by which GRK4 affects HepG2 cells using quantitative proteomics and targeted proteomics, which can provide a prospective translational viewpoint.

## 2 Materials and methods

### 2.1 Cell culture and lentiviral infection

HEK293T and HepG2 cells were acquired from the Chinese Academy of Sciences' Cell Bank (Shanghai, China). The cells were cultured in DMEM (Corning, USA) containing 10 % heat-inactivated bovine serum (sourced from the Ausbian serum family) and supplemented with puromycin (10 μg/mL) at 37 °C in 5 % CO_2_ atmosphere. The full-length human GRK4 (NM_182982) was synthesized by Shanghai GeneChem Co., Ltd. (Shanghai, China), and the GRK4-overexpressing lentivirus (LV-GRK4) and the negative control lentivirus were packaged by Shanghai GeneChem Co., Ltd. (Shanghai, China).

### 2.2 Validation of GRK4 expression

GRK4 mRNA and protein levels in the virus-infected HepG2 cell populations were measured via quantitative real-time polymerase chain reaction (PCR) and immunoblotting, following a previously described method [16]. To summarize, total RNA was extracted using TRIzol (Invitrogen, Waltham, MA, USA), and cDNA was synthesized using the PrimeScript RT Reagent Kit (Takara, Dalian, China) following the manufacturer's instructions. The primer sequences used for GRK4 were the forward, 5′-CAAGCAACCGATAGGAAGACG-3′, and the reverse, 5′-TCTGTCACAACATCTGGAGGTAT-3′; and the primer sequences used for GAPDH were the forward, 5′-TGACTTCAACAGCGACACCCA-3′, and the reverse, 5′-CACCCTGTTGCTGTAGCCAAA-3′.

Real-time PCR was carried out in an Applied Biosystems 7500 Fast Real-Time System using SYBR Green PCR Master Mix (Applied Biosystems, Wakefield, RI, USA) with the following PCR parameters: 95 °C for 5 min, then 40 cycles at 95 °C for 15 s, 60 °C for 15 s, and 72 °C for 15 s. GAPDH was employed as an internal control, and the fluorescence threshold value was computed to indicate the relative mRNA expression.

The immunoblotting procedure utilized to validate the expression of GRK4 [16] was carried out as follows: Proteins were extracted and denatured, separated under sodium dodecyl sulfate polyacrylamide gel electrophoresis, and then transferred to a membrane that was blocked before immunoblotting. Next, the primary antibodies (Rabbit GRK4,1:1000, HPA057023, Sigma-Aldrich, Laramie, WY, USA; Mouse GAPDH, sc-322331:1000, Santa Cruz Biotechnology, Dallas, TX, USA) and the secondary antibody (1:2000, anti-rabbit, anti-mouse, Santa Cruz) were incubated. Finally, the expression of GRK4 was developed using electrogenerated chemiluminescence.

### 2.3 The biological function phenotype in relation to cell proliferation, cycle, and apoptosis

The Cell Counting Kit-8 (CCK8) cell proliferation assay was carried out by first seeding virus-infected HepG2 cells into 96-well plates at a density of 1.5 × 10^3^ per well, after defining the GRK4 expression. Then, the cell proliferation features were tested with CCK8 (Dojindo Laboratories, Japan). The absorbance at 450 nm was measured every 24 h for 120 h to draw the cellular growth curve.

The cell cycle assay through flow cytometry (FCM) was performed by first seeding virus-infected HepG2 cells into 6-well plates at a density of 1 × 10^6^ per well and harvesting them for 24 h after being plated. Then, the cells were fixed with 80 % ethanol, stained with phosphate-buffered saline (PBS) containing 1 mg/mL propidium iodide and 10 mg/mL RNase A, and incubated at 37 °C. The cell cycle was detected using FCM and Cell Quest software (Becton, Dickinson, and Company, Franklin Lakes, NJ, USA).

The cell apoptosis assay was carried out after analyzing the distribution of cell apoptosis via FCM. Briefly, the apoptosis rate was evaluated using the Annexin V-APC/7-AAD Apoptosis Detection Kit according to the manufacturer's instructions (Lianke, China). The cells were seeded into 6-well plates (1 × 10^5^ cells/well) and collected after 24 h. The cells were then washed with PBS and resuspended in 100 μL binding buffer. Approximately 5 μL Annexin V-APC and 10 μL 7-AAD were added to the buffer and incubated at room temperature for 20 min in the dark. The cells were analyzed via FCM within 1 h.

### 2.4 Proteomics and bioinformatics analysis

A labelling experiment was performed with a tandem mass tag (TMT), following a previously described method [16], to examine the differential expression profile of HepG2 cells with and HepG2 cells without GRK4. Briefly stated, cells were collected, the total proteins were extracted using a filter-aided sample preparation, and peptides were tagged using the TMT reagent in accordance with the manufacturer's instructions (Thermo Fisher Scientific, Waltham, MA, USA). The TMT-labelled peptides were mixed and fractionated through reversed-phase chromatography with the Agilent 1260 Infinity II HPLC and evaluated through liquid chromatography–mass spectrometry (LC-MS/MS). A Q Exactive Plus mass spectrometer (Thermo Fisher Scientific, USA) was used for all LC-MS studies.

### 2.5 Parallel reaction monitoring analysis

Each sample (2 μg) was collected, separated through nano-LC, and subjected to online electrospray tandem mass spectrometry (Easy LLC, Q-Exactive, Thermo Fisher Scientific). Buffers A and B consisted of 0.1 % formic acid and 0.1 % for acetonitrile acetate (80 % acetonitrile), respectively. The samples were separated at 300 nL/min in the analysis column (Thermo Fisher Scientific, Acclaim PepMap RSLC 50 μm × 15 cm, nano viper, P/N164943) with a nonlinear gradient: 0 min–1 min, 2%–8%; 1 min–46 min, 8%–28 %; 46 min–56 min, 28%–40 %; 56 min–57 min, 40%–90 %; and 57 min–60 min, 90 %. The MS parameters were as follows: (1) Full-MS: scan range (*m*/*z*) = 350–1500; resolution = 60,000; automatic gain control (AGC) target = 1e6; maximum injection time = 50 ms; (2) PRM: resolution = 15,000; AGC target = 1e5; maximum injection time = 50 ms; loop count = 14; isolation window = 1.6 *m*/*z*; normalized collision energies = 27 %. All samples were instrumentally analyzed in triplicates. The isolation list was screened based on differentially expressed proteins (DEPs) that were related to relevant biological functions and pathways.

The data were processed using Proteome Discoverer 2.2, Mascot 2.6, and the UniProt database (Uniprot_HomoSapiens_20386_20180905, http://www.uniprot.org). When screening for differentially expressed proteins (DEPs), the filtering threshold was set as a fold-change (FC) of ≥1.2 or ≤0.83, and a *P*-value of <0.05 indicated statistical significance. The false discovery rate (FDR) for peptides and proteins was set at 1 %.

### 2.6 Statistical analysis

All acquired results were presented as the mean ± standard error of at least three separate experiments. The statistical analysis was carried out using IBM SPSS Statistics version 26.0 (SPSS, Chicago, USA). An independent-sample *t*-test was used to compare differences between the normal control group (NC group) and the GRK4 overexpression treatment group (OE group). A *P*-value of less than 0.05 was considered statistically significant.

## 3 Results

### 3.1 Identification of GRK4 expression in HepG2 cells

**Fig. 1:**
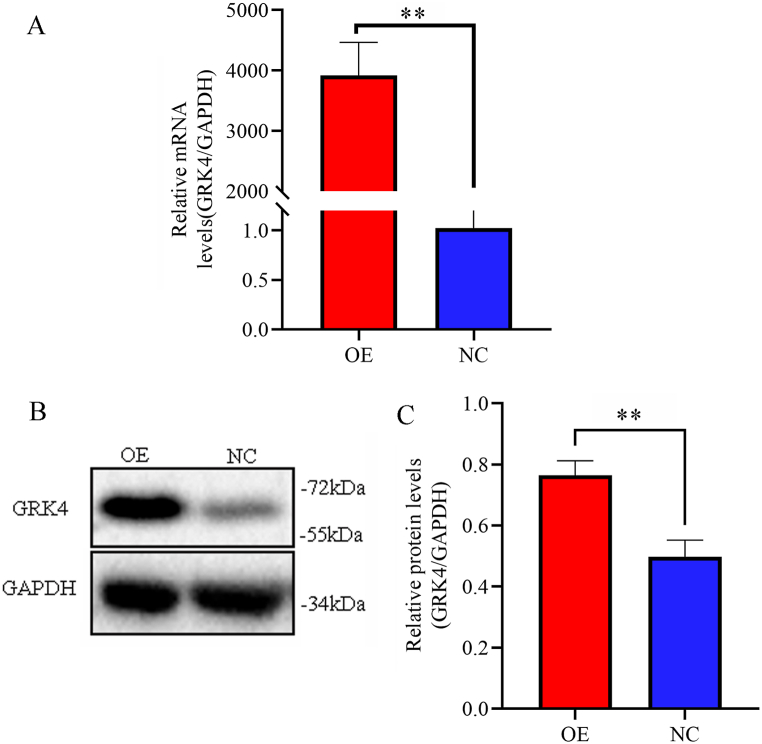
**Validation of GRK4 in lentivirus-infected HepG2 cells via fluorescence-based quantitative real-time PCR and immunoblotting.** (A) Expression of GRK4 mRNA, **p < 0.01. (B) GRK4 protein expression in infected cells assessed using immunoblotting with a GRK4 antibody. (C) The relative quantitative proteomics analysis of the GRK4 protein，**p < 0.01. Each experiment was repeated in triplicate. PCR, polymerase chain reaction.

To improve the comparability performance, lentivirus-infected HepG2 cells were employed as the normal control (NC group). The cells were examined and collected 72 h after infection. Real-time PCR and immunoblotting were used to identify the GRK4 expression levels in the two groups. The cells in the OE group expressed GRK4 exclusively (Fig. 1A and B, S1B，and 1C).

### 3.2 The cell biological phenotype in relation to cell proliferation, cycle, and apoptosis

**Fig. 2:**
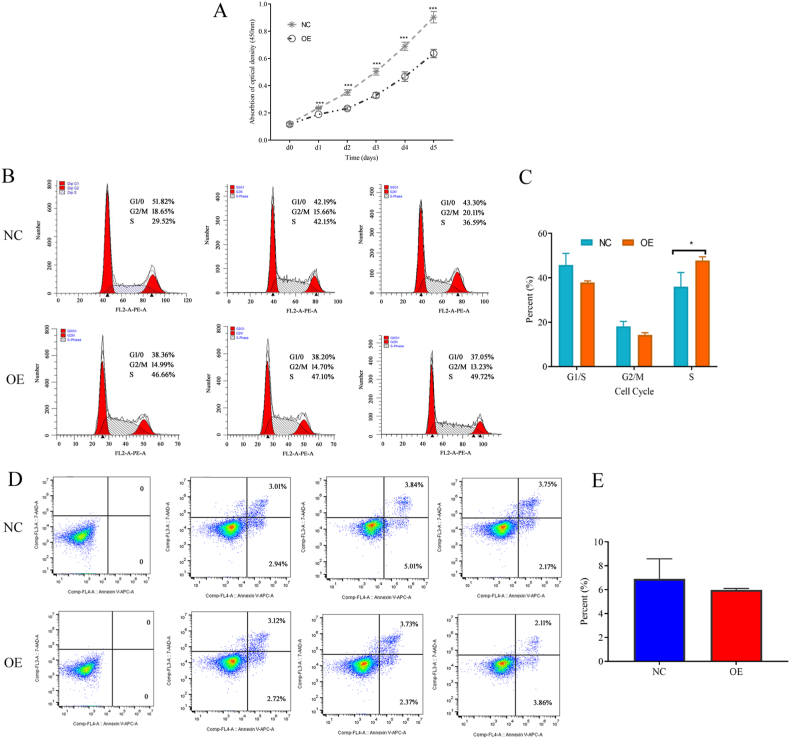
**The biological phenotype of HepG2 cells with and without GRK4 overexpression.** (A), an assay of the cell growth curve using the CCK8 test with the absorbance value set at 450 nm. The cells were harvested every 24 h for 120 h after being seeded in 96-well plates. The assay was conducted in triplicate. The statistical significance of the absorbance at 450 nm at d1–d5 was evaluated using an independent-sample *t*-test analysis, ***p < 0.001. (B) Distribution of the different phases of HepG2 cells via FCM using propidium iodide staining. (C) Mean distribution of the difference in cell cycles using the *t*-test, *p < 0.05. The test was replicated in triplicate. (D) Distribution of cell apoptosis via FCM using Annexin V-APC/7-AAD. (E) Mean distribution of cell apoptosis using the *t*-test, replicated in triplicate. FCM, flow cytometry.

HepG2 cells proliferated in both groups, but cells in the NC group without GRK4 expression expanded exponentially, whereas cells in the OE group with GRK4 expression grew more slowly (*P* < 0.001) (Fig. 2A).

HepG2 cells with overexpressed GRK4 exhibited a significant increase in population at the S-phase (47.83 ± 1.65 %) compared to cells in the NC group (36.09 ± 6.33 %, *P* < 0.05). The percentage of cells in the G1/0 (37.8 ± 0.71 % vs. 45.77 ± 5.26 %, *P* > 0.05) and G2/M (14.3 ± 0.94 % vs. 18.11 ± 2.27 %, *P* > 0.05) phases did not differ between the OE and NC groups (Fig. 2B and C).

Annexin V and APC staining kits were used to measure HepG2 cell apoptosis, and Fig. 2D and E demonstrate the proportion of certain cell types at various stages of apoptosis. Unfortunately, the apoptosis rate was not statistically significant (*P* > 0.05) in the OE and NC groups: 5.97 ± 0.08 % vs. 6.91 ± 0.97 %, respectively.

### 3.3 Quantitative analysis of the effect of the proteome of GRK4 on HepG2 cells

**Fig. 3:**
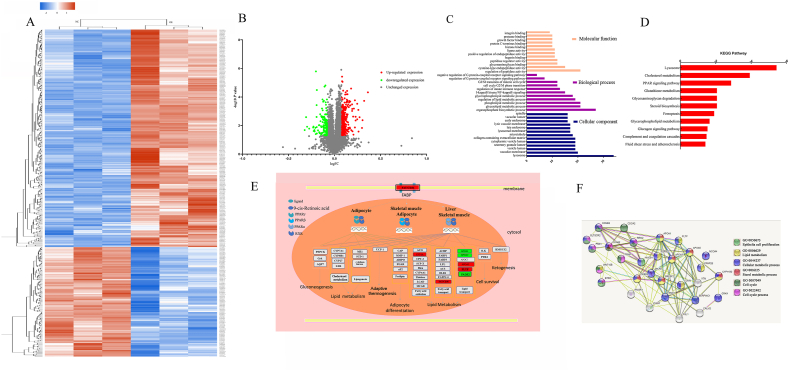
**Functional annotation and informative analysis of DEPs**. (A) Cluster heatmap of DEPs. (B) Volcano plot of DEPs and identified proteins. Filter threshold of DEPs set as a n FC above 1.2 or below 0.83; *p* < 0.05. The x-axis represents the log2 FC, and the y-axis represents the -log10 *p* value. The grey points indicate fully quantified, unaltered proteins; the green points and red points indicate downregulated and upregulated DEPs, respectively. (C) Kyoto Encyclopedia of Genes and Genomes pathway enrichment of DEPs using Cytoscape 3.9.1 with *p* < 0.01. (D) Enriched function through gene ontology using Cytoscape 3.9.1; *p* < 0.01. (E) Exhibition of PPAR pathway enriched using DEPs: the red rectangle indicates upregulated DEPs; the green rectangle indicates downregulated DEPs. (F) Visualization of the protein–protein interaction network and biological process network by interested DEPs using String 11.5 with *p* < 0.01; the heterogenous color indicates a biological process. DEPs, differentially expressed proteins; FC, fold-change; PPAR, peroxisome proliferator-activated receptor. (For interpretation of the references to color in this figure legend, the reader is referred to the Web version of this article.)

Protein mass spectrometry analysis obtained a total of 65,116 distinct peptides, and 7006 proteins were discovered after database matching. Following a quantitative analysis of the identified proteins, 403 DEPs were filtered using the filtering parameters (FC ≥ 1.2 or ≤ 0.83 and *P* < 0.05), as seen in Fig. 3A. As indicated in Fig. 3B and Tables S1 and 268 proteins showed upregulated expression, whereas 135 proteins exhibited downregulated expression. These proteins were employed to advance gene ontology annotation and function enrichment, including the molecular function (MF), biological process, and cellular component (CC) (Fig. 3C and Table S2). The MF was responsible for the regulation of peptidase activity, cysteine-type endopeptidase activity, growth factors, glycosaminoglycans, heparin, and histones (Table S2A). An intricate network structure was created based on the interactions among all of the molecules in the system. On the other hand, the DEPs participated in the regulation of several biological processes, including glycerolipid metabolism, phospholipid metabolism, lipid metabolic process, NF–B signaling, the cell cycle, and the innate immune response, etc. GRK4, SLC39A14, STMN1, and 14-3 protein (YWHAB) participated in GPCR signaling pathways and regulatory mechanisms (Table S2B). It has been demonstrated that CCNA2, CCNB2, and other proteins are involved in cell cycle regulation (Table S2B). According to the analysis, the majority of the DEPs were discovered in the lysosomes and vacuolar membrane (Table S2C). Meanwhile, DEPs were used to enrich the Kyoto Encyclopedia of Genes and Genomes signaling pathway (Fig. 3D and Table S2D). As depicted in Table S2D, the current study revealed that CD36, FADS2, APOA1, APOA2, and APOA5 are engaged in the signaling pathways of peroxisome proliferator-activated receptors (PPARs), as well as a variety of metabolic activities, including glycerophospholipid metabolism and cholesterol metabolic processes (Fig. 3D, E, and 3F). Additionally, in this study, NCOA4, PRNP, SLC39A14, and TFRC were found to be involved in programmed iron death (Table S2D). Lastly, a protein–protein interaction study was conducted using the DEPs associated with cell proliferation, cycle, and metabolism (Fig. 3F). This proved that the DEPs maintained a flexible connection as well as interaction within the biological processes in which they were involved. The MS proteomics data have been deposited into the Proteome Xchange Consortium (http://proteomecentral.proteomexchange.org) via the iProX partner repository [17,18], with the dataset identifier PXD039951.

### 3.3 Development of high-throughput PRM for the analysis of GRK4 on HepG2 cells

**Fig. 4:**
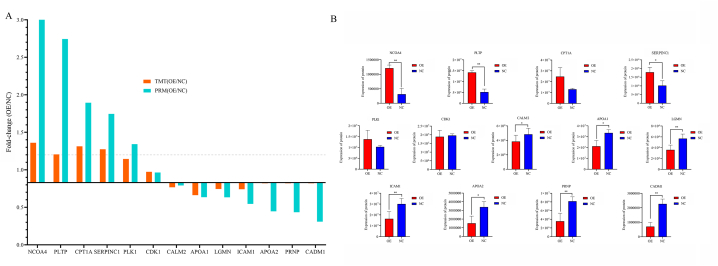
**Quantitative analyses of DEPs.** (A) Qualitative analysis of 22 proteins associated with cell proliferation, cycle, and metabolism using a validation PRM; the results of the PRM analysis were consistent with the TMT results. The longitudinal axis, where the solid line represents 0.83 and the dotted line represents 1.2. Proteins located above the dashed line (FC > 1.2) were upregulated, and those located below the solid line (FC < 0.83) were downregulated. Proteins between the solid and dashed lines were expressed without appreciable changes. (B) The expression of interested target proteins was verified via the PRM. *p < 0.05, **p < 0.01. FC, fold-change; PRM, parallel response monitor; TMT, tandem mass tag.

To validate the proteomics findings, several proteins were selected for a PRM experiment. The proteins associated with the PPAR pathway, proliferation, cell cycle, and metabolism—including upregulated and downregulated DEPs—as well as unchanged proteins were the criteria used in the filtering set (Table S3). According to the PRM, there were thirteen downregulated proteins; two unaltered proteins, PLK1 (*P* = 0.217) and CDK1 (*P* = 0.765); and six upregulated proteins. A major differential protein (CPT1A, *P* = 0.07), which was expressed at a level that was almost at the threshold for statistical significance, was also elevated (Fig. 4A and Table S4). The results were comparable to the findings of the TMT study. Fig. 4B illustrates the expressions of part-filtered DEPs and unchanged proteins, which were validated by PRM (NCOA4, PLTP, CPT1A, SERPINC1, PLK1, CDK1, CALM2, APOA1, LGMN, ICAM1, APOA2, PRNP, and CADM1). Validation of the PRM demonstrated a clear correlation between GRK4's inhibitory effects on HCC cells and DEPs from the PPAR pathway.

## 4 Discussion

HCC is a cancer with a dismal prognosis and complicated pathogenic processes involving genetic and epigenetic abnormalities. The goal of HCC research is to uncover potential biomarkers and therapeutic targets associated with its pathogenesis.

The current study found that prolonged lentivirus-mediated overexpression of GRK4 inhibited the development of HepG2 cells and caused cell cycle arrest in the S stage. In our recent work, after transiently transfecting GRK4 into HEK293 cells and separating the cells with positive and negative GRK4 expression via FCM, it was discovered that HEK293 ^GRK4+^ cells showed proliferation retardation and the G1 stage cell cycle in comparison to HEK293 ^GRK4−^ cells [16]. GRK4 phosphorylated GPCR that had been stimulated by extracellular ligands and used β-arrestin to prevent GPCR from attaching to the G protein, which caused desensitization or internalization of GPCR. GKR4 then ended the negative regulation of GPCR [5,8,19]. Four splice isomers (α/β/γ/δ) distinguish the distinct form of GRK4 in humans [20] and are associated with the heterogeneous expression of distinct isomers of GRK4. According to an ovarian cancer study, GRK4α/β expression in nonmalignant granulosa cells is six times greater than that of malignant granular cells, while GRK4γ/δ expression is two times greater than that of nonmalignant tumor cells. The uncoupling of luteinizing hormone/villin-stimulating gonadotropin receptors can be enhanced by spliced GRK4 isoforms. Reduced GRK4α/β expression has the potential to cause kinase activity impairment, FSHR isotype uncoupling, and receptor desensitization [8,21]. Finally, consequent receptor activation has been shown to cause ovarian granulosa cell cancer [20]. The influence of GRK4 was confirmed by the existence of hyperfunction thyroid nodules [22]. However, GRK4's effect on breast cancer completely contradicted the above findings [[12], [13], [14]]. Initially, more than one GRK4 isoform (GRK4β/δ, GRK4α/β/δ, and GRK4β/γ/δ) were detected in tumor tissues obtained from the same patient with breast cancer [13]. In addition to being the source of hypertension, persistent activation of the GRK4 single nucleotide polymorphism genotype (A142 V or R65L, A486V) was often seen in breast cancer cell lines [12,14,23]. Consequently, GRK4 was discovered to be an oncogene in breast cancer and a co-regulator of hypertension [12,14]. GRK4 stimulated the growth of breast cancer cells (MCF-7) via the MAPK signaling pathway [13,14]. In the present study, GRK4 inhibited the growth of HepG2 cells, which suggested that GRK4 might perform two heterogeneous functions in different types of tumor cells. Moreover, non-visual GRK member investigations have shown that GRK5 and GRK3 act completely differently in a variety of tumor types [[24], [25], [26], [27]]. In particular, GRK5's subcellular location affects both its inhibitory and promotive actions; therefore, the type of tumor is not the only factor affecting them. Research has shown that whereas GRK5 expression in the cytoplasm suppresses tumor growth, its presence in the nucleus promotes the formation of tumors [24]. Unlike the main location of GRK5 expression in the nuclear membrane, previous research has shown that the main location of GRK4 expression in HCC was cell plasma [15], although the detailed mechanism of GRK4 regulation of liver cancer remains to be elucidated.

The current study used proteomics techniques to investigate the mechanism of GRK4 regulation of hepatoma cells. Numerous proteins, most of which are involved in the cell cycle, proliferation, metabolic processes, and PPAR pathway, were shown to be impacted by GRK4 in terms of their expression. The PPAR pathway has a proven link to lipid metabolism, adipogenesis, metabolic balance, inflammation, and tumor development [28]. This investigation did not observe any changes in PPAR expression that were induced by GRK4. It was revealed that GRK4 and PPARs are significant regulators of hypertension and HCC [6,15,[29], [30], [31], [32]]. Although there has been no evidence that GRK4 impacts the PPAR, it was discovered that GRK2, a member of the GRK family, might be the cause of hypertension due to overexpression and PPAR downregulation [33]. GRK2 regulation might play a role in PPAR ligand-mediated blood pressure reduction, which suggests that GRK4 and PPARs might have an indirect relationship [33]. Critical nodes in the PPAR pathway—APOA1, APOA2, PLTP, and APOA5—were identified in the current investigation. Throughout the progression of HCC, it was demonstrated that APOA1 interacts with the other nodes and plays a crucial regulatory role in lipid metabolism and the MAPK pathway, although the exact mechanism must be validated using a large cohort of human HCC samples [34]. APOA1 and APOA2 were the main components of high-density lipoprotein (HDL); the former helped move cholesterol from tissues to the liver in reverse, whereas the latter stabilized the HDL structure and affected HDL metabolism [28,35,36]. APOA1 and APOA2 were significantly expressed during the early stage and advanced stage of HCC [28,[37], [38], [39]]. By inhibiting the MAPK pathway, APOA1 alone delayed the cell cycle and decreased the growth of HCC cells [40]. Nevertheless, low levels of APOA1 in HCC patients were associated with poor prognoses [41,42]. Such adverse outcomes might be brought on by a tumor load that damages the liver severely or by hepatic function loss after radiotherapy, chemotherapy, or surgery. In the current study, GRK4 caused aberrant expression of APOA1 and APOA2 in HepG2 cells, and PRM confirmed that APOA1 and APOA2 were downregulated, while the cells showed inhibited growth and cycle arrest. Diverse GRK4 expression was seen in HCC tissues in the previous investigation, and individuals with HCC benefited from high GRK4 expression in the tumor [15].

The current study consolidated GRK4's negative control function in HepG2 cells. Concomitant with the cellular phenotype change was an abnormal expression of a series of proteins associated with metabolic and PPAR pathways such as APOA1, APOA2, etc. Nevertheless, this study had the following limitations: It was an exploratory study restricted to HepG2 cells, so future research on the regulation of HCC by GRK4 will require the use of expanded hepatoma cell lines. The initial analysis of the mechanism only assessed quantitative proteomics and targeted proteomics; therefore, further research is needed to determine the precise mechanism of GRK4 in regulating HCC.

## 5 Conclusion

According to the current study, GRK4 led to S-phase cell cycle arrest and inhibited the proliferation of HepG2 cells. Variations in related proteins involved in cell proliferation, cycle, and lipid metabolism were found via quantitative proteomic analysis. PRM confirmed the expression of DEPs, and the PPAR pathway may have had a role in the negative regulation of HepG2 cells by GRK4; more studies are needed to determine the precise process.

## Funding

Not applicable.

## Data availability statement

The mass spectrometry proteomics data have been deposited to the Proteome Xchange Consortium (http://proteomecentral.proteomexchange.org/cgi/GetDataset?ID=PXD039951) via the iProX partner repository with the dataset identifier (https://www.iprox.cn/page/project.html?id=IPX0005807000).

## CRediT authorship contribution statement

**Yunxiu Luo:** Writing – review & editing, Writing – original draft, Formal analysis, Data curation, Conceptualization. **Jing Yang:** Methodology, Formal analysis. **Yan Wang:** Formal analysis, Data curation.

## Declaration of competing interest

The authors declare that they have no known competing financial interests or personal relationships that could have appeared to influence the work reported in this paper.

## Figures and Tables

**figs1:**